# Supplementary material for: Associations of a plant-centered diet and lung function across early to mid-adulthood: The CARDIA Lung Study
Source: Respir Res. 2024 Mar 11;25:122. doi: 10.1186/s12931-023-02632-x (PMC10926674; doi:10.1186/s12931-023-02632-x)
Supplement: Supplementary file 1 — Additional file 1: Table S1. Baseline characteristics for participants excluded and included. [file 12931_2023_2632_MOESM1_ESM.docx]

| **Table S1. Baseline characteristics for participants excluded and included** | |  |  |
| --- | --- | --- | --- |
|  | Excluded (n=1327) | Included (n=3787) | P-value^a^ |
| APDQS, mean±SD | 59.9±12.2 | 63.5±13.1 | <0.001 |
| Age Y0, mean±SD, y | 24.2±3.7 | 25.1±3.6 | <0.001 |
| Female, no (%) | 637 (48.0) | 2150 (56.8) | <0.001 |
| Self-identified race, no (%) |  |  |  |
| Black | 845 (63.7) | 1792 (47.3) | <0.001 |
| White | 482 (36.3) | 1995 (52.7) |  |
| Maximal educational attainment, mean±SD, grades^b^ | 14±2.4 | 15.7±2.6 | <0.001 |
| Study center, no (%) |  |  |  |
| Birmingham | 296 (22.3) | 882 (23.3) | <0.001 |
| Chicago | 272 (20.5) | 836 (22.1) |  |
| Minneapolis | 427 (32.2) | 975 (25.8) |  |
| Oakland | 332 (25.0) | 1094 (28.9) |  |
| Height, mean±SD, cm | 171±9 | 170.3±9.5 | 0.22 |
| BMI, mean±SD, kg/m^2^ | 24.5±5.3 | 24±5 | 0.80 |
| Smoking, no (%) |  |  |  |
| Never | 609 (46.3) | 2247 (59.7) | <0.001 |
| Former | 155 (11.8) | 521 (13.9) |  |
| Current | 552 (42.0) | 994 (26.4) |  |
| Pack-years smoking at Y0, mean±SD, pack-years | 2.7±4.8 | 2.1±4.3 | <0.001 |
| Pack-years smoking throughout Y20, mean±SD, pack-years | 5.9±9.4 | 5.1±9.4 | 0.003 |
| Total energy intake, mean±SD, kcal | 3075±1495 | 2738±1269 | <0.001 |
| Physical activity, mean±SD, EU^c^ | 433.2±321.7 | 415.5±292.9 | 0.07 |
| Cardiorespiratory fitness, mean±SD, treadmill time, second | 572±174 | 591.5±170.9 | 0.001 |
| History of asthma, no (%) | 70 (5.3) | 179 (4.7) | 0.32 |
| Emphysema at Y25, no (%) | 45 (3.6) | 125 (3.5) | 0.82 |
| ^a^ Evaluated with chi-square tests for categorical variables and ANOVA for continuous variables. | | |  |
