# Supplementary material for: Associations of a plant-centered diet and lung function across early to mid-adulthood: The CARDIA Lung Study
Source: Respir Res. 2024 Mar 11;25:122. doi: 10.1186/s12931-023-02632-x (PMC10926674; doi:10.1186/s12931-023-02632-x)
Supplement: Supplementary file 2 — Additional file 2: Table S2. Association between year 0 APDQS and annual changes in pulmonary function measures. [file 12931_2023_2632_MOESM2_ESM.docx]

| **Table S2. Association between year 0 APDQS and annual changes in pulmonary function measures ^a^** | | |
| --- | --- | --- |
|  | **Estimated slopes per 1 SD higher updated average APDQS ^b^** | |
|  | ***ß±SE*** | **P-value** |
| **FVC annual absolute change, ml** |  |  |
| Mean±SD | -35.8±20 |  |
| MV model ^c^ | 0.84±0.38 | 0.03 |
| MV model + cardiorespiratory fitness ^d^ | 0.35±0.39 | 0.37 |
| MV model + current asthma ^d^ | 0.85±0.38 | 0.02 |
|  |  |  |
| **FEV_1_ annual absolute change, ml** |  |  |
| Mean±SD | -34.4±15.8 |  |
| MV model ^c^ | 0.19±0.30 | 0.51 |
| MV model + cardiorespiratory fitness ^d^ | -0.16±0.30 | 0.61 |
| MV model + current asthma ^d^ | 0.23±0.30 | 0.44 |
|  |  |  |
| **FEV_1_/FVC ratio, 100*annual absolute change in the ratio** |  |  |
| Mean±SD | -0.31±0.25 |  |
| MV model ^c^ | -0.006±0.005 | 0.22 |
| MV model + cardiorespiratory fitness ^d^ | -0.007±0.005 | 0.16 |
| MV model + current asthma ^d^ | -0.006±0.005 | 0.22 |
| ^a^ (Year 30 FVC - peak FVC)/( 30 - peak year). Other measures were calculated in the same way. If measurements at Year 30 were not available, Year 20 data were used, with denominator (20 – peak year). | | |
| ^b^ 1 SD = 13. |  |  |
| ^c^ Multivariable-adjusted linear regression model. APDQS a continuous variable, the average of Y0, Y7, and Y20, with infrequent missing Y7 or Y20 last value carried forward. The regression is adjusted for peak pulmonary function variable (depending on outcome of interest), age squared, sex, race (Black and White), center (Birmingham, Chicago, Minneapolis, and Oakland), maximal educational attainment, baseline height, averaged total energy intake, averaged BMI, and life-time pack years of smoking. | | |
