# Supplementary material for: Associations of a plant-centered diet and lung function across early to mid-adulthood: The CARDIA Lung Study
Source: Respir Res. 2024 Mar 11;25:122. doi: 10.1186/s12931-023-02632-x (PMC10926674; doi:10.1186/s12931-023-02632-x)
Supplement: Supplementary file 3 — Additional file 3: Table S3. Multivariable-adjusted HRs (95% CIs) of incident obstructive lung disease according to quintiles of the APDQS for current, former, and never smokers. [file 12931_2023_2632_MOESM3_ESM.docx]

| **Table S3. Multivariable-adjusted HRs (95% CIs) of incident obstructive lung disease according to quintiles of the APDQS for current, former, and never smokers, N=3,487** | | | | | | | |
| --- | --- | --- | --- | --- | --- | --- | --- |
|  | **APDQS** | | | | |  |  |
|  | **Quintile 1** | **Quintile 2** | **Quintile 3** | **Quintile 4** | **Quintile 5** | **Per 1 SD higher APDQS ^a^** | **P for trend ^b^** |
| **Never smokers (n=1,842)** |  |  |  |  |  |  |  |
| APDQS median | 51.6 | 59.7 | 66.0 | 73.0 | 82.0 |  |  |
| Unadjusted cumulative incidence % (n/N) ^c^ | 11.5 (43/374) | 7.9 (28/353) | 7.6 (29/381) | 11 (43/390) | 13.7 (47/344) |  |  |
| Unadjusted HR | 1 (ref) | 0.79  (0.49-1.26) | 0.73  (0.45-1.17) | 1.04  (0.68-1.61) | 1.28  (0.84-1.95) | 1.12  (0.96-1.30) | 0.16 |
| MV model HR ^d^ | 1 (ref) | 0.76  (0.47-1.23) | 0.66  (0.40-1.10) | 0.90  (0.55-1.50) | 1.05  (0.61-1.79) | 1.03  (0.85-1.26) | 0.75 |
| MV model HR+ cardiorespiratory fitness ^e^ | 1 (ref) | 0.73  (0.45-1.18) | 0.65  (0.40-1.08) | 0.86  (0.52-1.44) | 0.96  (0.55-1.68) | 1.00  (0.82-1.22) | 0.99 |
| MV model HR + current asthma ^e^ | 1 (ref) | 0.78  (0.48-1.26) | 0.67  (0.41-1.11) | 0.89  (0.54-1.48) | 1.01  (0.59-1.73) | 1.01  (0.83-1.24) | 0.89 |
| **Former smokers (n=995)** |  |  |  |  |  |  |  |
| APDQS median | 53.0 | 60.0 | 66.0 | 73.3 | 82.7 |  |  |
| Unadjusted cumulative incidence % (n/N) ^c^ | 16 (19/119) | 10.1 (15/148) | 8.9 (19/214) | 13.9 (32/230) | 16.9 (48/284) |  |  |
| Unadjusted HR | 1 (ref) | 0.66  (0.32-1.33) | 0.67  (0.36-1.28) | 1.04  (0.58-1.85) | 1.09  (0.63-1.91) | 1.17  (0.96-1.41) | 0.12 |
| MV model HR ^d^ | 1 (ref) | 0.63  (0.31-1.28) | 0.65  (0.34-1.27) | 0.95  (0.50-1.79) | 0.97  (0.50-1.87) | 1.12  (0.88-1.43) | 0.35 |
| MV model HR+ cardiorespiratory fitness ^e^ | 1 (ref) | 0.61  (0.30-1.26) | 0.64 (0.33-1.  25) | 0.91  (0.48-1.73) | 0.99  (0.51-1.95) | 1.14  (0.89-1.46) | 0.30 |
| MV model HR + current asthma ^e^ | 1 (ref) | 0.61  (0.30-1.25) | 0.64  (0.33-1.24) | 0.90  (0.48-1.70) | 0.94  (0.49-1.82) | 1.12  (0.88-1.43) | 0.35 |
| **Current smokers (n=650)** |  |  |  |  |  |  |  |
| APDQS median | 52.0 | 59.0 | 66.0 | 72.3 | 81.7 |  |  |
| Unadjusted cumulative incidence % (n/N) ^c^ | 25.7 (45/175) | 21.1 (38/180) | 21.4 (28/131) | 21.1 (20/95) | 23.2 (16/69) |  |  |
| Unadjusted HR | 1 (ref) | 0.94  (0.61-1.45) | 0.81  (0.49-1.32) | 0.86  (0.51-1.47) | 1.04  (0.60-1.79) | 0.97  (0.79-1.18) | 0.73 |
| MV model HR ^d^ | 1 (ref) | 0.93  (0.60-1.45) | 0.91  (0.54-1.55) | 1.06  (0.58-1.91) | 1.12  (0.58-2.18) | 0.99  (0.78-1.27) | 0.96 |
| MV model HR+ cardiorespiratory fitness ^e^ | 1 (ref) | 0.93  (0.59-1.46) | 0.91  (0.53-1.55) | 0.96  (0.52-1.78) | 1.17  (0.59-2.33) | 0.99  (0.77-1.28) | 0.96 |
| MV model HR + current asthma ^e^ | 1 (ref) | 0.92  (0.59-1.42) | 0.87  (0.52-1.48) | 1.07  (0.59-1.93) | 1.10  (0.57-2.13) | 0.99  (0.78-1.26) | 0.92 |
| ^a^ 1 SD was 13. |  |  |  |  |  |  |  |
| ^b^ Derived by testing a continuous APDQS variable in the model. | | | | | | |  |
| ^c^ Cumulative incidence of obstructive lung disease from Year 2 through Year 30 | | | | | | | |
| ^d^ Cox proportional-hazards regression model adjusted for age, sex, race (Black and White), center (Birmingham, Chicago, Minneapolis, and Oakland), maximal educational attainment, baseline height, averaged total energy intake (Years 0, 7, 20), averaged BMI (Years 0, 2, 5, 7, 10, 15, and 20), and life-time pack years of smoking (Years 0, 2, 5, 7, 10, 15, and 20). | | | | | | | |
| ^e^ Time-updated covariates were used (Years 0, 2, 5, 7, 10, 15, and 20). | | | | | | |  |
